# Genetic Artificial Intelligence in Gastrointestinal Disease: A Systematic Review

**DOI:** 10.3390/diagnostics15172227

**Published:** 2025-09-02

**Authors:** Kwang-Sig Lee, Eun Sun Kim

**Affiliations:** 1 AI Center, Korea University Anam Hospital, Seoul 02841, Republic of Korea; 2 Department of Gastroenterology, Korea University Anam Hospital, Seoul 02841, Republic of Korea

**Keywords:** gastrointestinal disease, artificial intelligence, genetic data, single nucleotide polymorphism

## Abstract

**Background/Objectives**: The application of predictive and explainable artificial intelligence to bioinformatics data such as single nucleotide polymorphism (SNP) information is attracting rising attention in the diagnosis of various diseases. However, there are few reviews available on the recent progress of genetic artificial intelligence for the early diagnosis of gastrointestinal disease (GID). The purpose of this study is to complete a systematic review on the recent progress of genetic artificial intelligence in GID. **Methods**: The source of data was ten original studies from PubMed. The ten original studies were eligible according to the following criteria: (participants) the dependent variable of GID or associated disease; (interventions/comparisons) artificial intelligence; (outcomes) accuracy, the area under the curve (AUC), and/or variable importance; a publication year of 2010 or later; and the publication language of English. **Results**: The performance outcomes reported varied within 79–100 for accuracy (%) and 63–98 for the AUC (%). Random forest was the best approach (AUC 98%) for the classification of inflammatory bowel disease with 13 single nucleotide polymorphisms (SNPs). Similarly, random forest was the best method (R-square 99%) for the regression of the gut microbiome SNP saturation number. The following SNPs were discovered to be major variables for the prediction of GID or associated disease: rs2295778, rs13337626, rs2296188, rs2114039 (esophageal adenocarcinoma); rs28785174, rs60532570, rs13056955, rs7660164 (Crohn’s disease early intestinal resection); rs4945943 (Crohn’s disease); rs316115020, rs316420452 (calcium metabolism); rs738409_G, rs2642438_A, rs58542926_T, rs72613567_TA (steatotic liver disease); rs148710154, rs75146099 (esophageal squamous cell carcinoma). The following demographic and health-related variables were found to be important predictors of GID or associated disease besides SNPs: age, body mass index, disease behavior, immune cell type, intestinal microbiome, MARCKS protein, smoking, and SNP density/number. No deep learning study was found even though deep learning was used as a search term together with machine learning. **Conclusions**: Genetic artificial intelligence is effective and non-invasive as a decision support system for GID.

## 1. Introduction

### 1.1. Gastrointestinal Disease

Gastrointestinal disease (GID) is a significant contributor to the global disease burden [1,2,3,4,5,6]. It encompasses the gastrointestinal tract, such as the esophagus, liver, stomach, small and large intestines, gallbladder, and pancreas [1]. GID is responsible for 8 million deaths worldwide each year [2] and 120 billion USD of total expenditure in the United States as of 2018 [3]. In Korea, GID ranked 8th among 21 disease groups in terms of disability-adjusted life years, with 1730 per 100,000 (5.9%) in 2015 [4]. The medical costs of GID in Korea amounted to 4 billion USD or 13% of all medical costs in the country in 2007 [5]. Various factors can contribute to the development of GID, including poor health behaviors like excessive stress, disrupted routine, insufficient exercise, a low-fiber diet, and a high-dairy diet. Unhealthy bowel habits, excessive anti-diarrheal/antacid medication, and pregnancy can also be causes of GID [6]. There are two types of GID: functional and structural. Functional GID is characterized by the normal appearance of the gastrointestinal tract but motility issues are revealed during medical examinations. Common examples include poisoning, nausea, irritable bowel syndrome, gastroesophageal reflux disease (GERD), gas, diarrhea, constipation, and bloating. Structural GID, on the other hand, involves abnormalities in both the appearance and motility of the gastrointestinal tract. Conditions such as strictures, stenosis, inflammatory bowel disease, hemorrhoids, diverticular disease, colorectal polyps, and colorectal cancers fall under this category. GID can be prevented through good health behaviors including regular colonoscopy screenings and good bowel habits [1,6].

### 1.2. Explainable Artificial Intelligence

Currently, the idea of artificial intelligence is receiving global attention. Artificial intelligence can be defined as “the capability of a machine to imitate intelligent human behavior” (the Merriam–Webster dictionary). It can be denoted that machine learning is a branch of artificial intelligence used “to extract knowledge from large amounts of data” [7]. Popular machine learning methods are support vector machine, random forest, naïve Bayesian predictor, decision tree, and artificial/deep neural networks (see [7] for a description of these methods). The validity of traditional study might be quite limited regarding the early diagnosis of disease, given that it uses logistic regression with an unrealistic assumption of *ceteris paribus*, i.e., “all the other variables staying constant”. In this context, the emerging literature employs artificial intelligence for the early diagnosis of disease, e.g., breast cancer [8], surgery [9], autism [10], cardiac arrest [11], weight loss [12], blood management [13], depression [14], brain disease [15], preterm birth [16], safe balance [17], neurodevelopmental delay [18], and gait recovery [19]. It is free from unrealistic assumptions of *ceteris paribus*. It presents the importance values and rankings of independent variables for the early diagnosis of the dependent variable.

Furthermore, the idea of explainable artificial intelligence is gaining great popularity at this point. Explainable artificial intelligence can be denoted as “artificial intelligence to identify major independent variables for the classification or regression of the dependent variable”, and it can be divided into four methods including random forest impurity importance, random forest permutation importance [20,21], machine learning accuracy importance, and Shapley additive explanations (SHAP) [22,23,24,25,26,27,28,29,30,31,32]. Random forest impurity importance is the node impurity decrease from the construction of a branch on a certain independent variable. It is an average over all trees in a random forest with the range of 0 and 1. Random forest permutation importance is the overall accuracy decrease from the permutation of data on the independent variable. It is an average over all trees in the random forest with a value of 0 to 1 [20,21]. Machine learning accuracy importance (an extended version of random forest permutation importance) is the accuracy decrease from the exclusion of data on the independent variable. The SHAP value of an independent variable for a participant is the difference between what machine learning predicts for the probability of the dependent variable (e.g., GID) with and without the independent variable [22,23,24,25,26,27,28,29,30,31,32].

### 1.3. Genetic Artificial Intelligence

Genetic artificial intelligence (AI) refers to “the application of artificial intelligence for bioinformatics data” [33,34,35]. Bioinformatics combines biology and informatics for the collection, analysis, and interpretation of genetic data. Bioinformatics data include (1) protein sequences consisting of 22 amino acids A, R, N, …, Y, V [proteomics]; (2) deoxyribonucleic acid (DNA) sequences consisting of four nucleotides A, C, G, and T [genomics]; (3) ribonucleic acid (RNA) sequences consisting of four nucleotides A, C, G, and U [genomics]. Here, a nucleotide includes a nitrogenous basis, a pentose sugar, and a phosphate group. In the central dogma of current bioinformatics, DNA sequences are transcribed into RNA sequences, which, finally, are transcribed into protein sequences. In other words, an amino acid of a protein can be expressed as RNA nucleotide triplets (codons) or their DNA counterparts, e.g., Ala (A) as GCU or GCC in Table 1 [36]. For example, insulin, which is a protein chain of 110 amino acids (MALWMR … LENYCN), can be expressed as its DNA counterpart of 110 nucleotide triplets (ATG GCC … TGC AAC). Here, amino acids M, A, C, and N correspond to nucleotide triplets ATG, GCC, TGC, and AAC, respectively. Popular bioinformatics databases are the GenBank [37], GeneCards [38], and UniProt [39].

**Figure 1:**
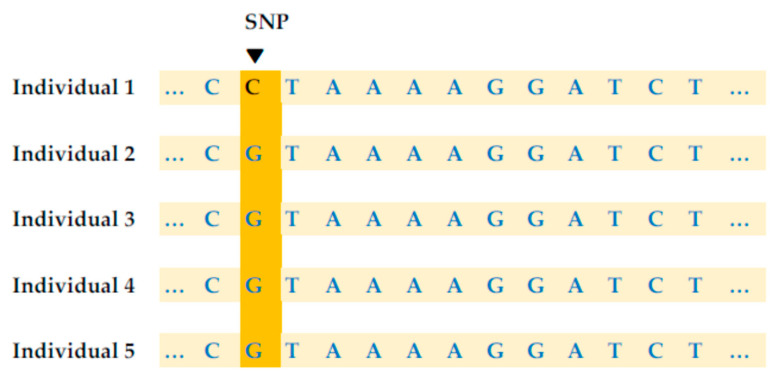
Single nucleotide polymorphism.

The human genome is often called “an ocean of information” with 3.2 billion bases. However, only 23,000 genes have been discovered. This accounts for just 3% of the human genome and the rest is still unknown to us. Replication is the quite effective and largely seamless process of copying the DNA sequence in a cell. But a certain error can happen in this process and the cell cannot restore itself completely on some occasions [40]. A single nucleotide polymorphism (SNP) is a one-letter place (A, T, C, or G) where an individual’s DNA sequence varies from other individuals’ DNA sequences [41]. For example, individual 1’s DNA sequence is different, as C, from other individuals’ DNA sequences, as G, in Figure 1 [40]. SNPs can serve as biomarkers, which distinguish those with disease from those without disease. A variation is considered to be an SNP when it occurs in 1% or more of a population. But this “1%” requirement is not universal [41]. An SNP happens after every 1000 nucleotides hence 4 million SNPs occur in the human genome. As addressed above, SNPs happen during the process of replication, and a thousand to a million SNPs occur every single day given that the process of replication never stops in the human genome [40,41]. SNPedia [42] is a common SNP database.

The application of predictive and explainable artificial intelligence for bioinformatics data such as SNP information is attracting rising attention in the diagnosis of various diseases, e.g., asthma [43], diabetes [44], hypertension [45], malaria [46], Parkinson’s diseases [47], schizophrenia [48], and viruses [49,50]. For example, a recent study used the random forest as a predictive and explainable artificial intelligence technique for the classification of asthma [43]. Data came from 128 participants with 176,288 SNPs enrolled in an open data source. The baseline approach (comparison) was the nearest neighbor while the innovation approach (intervention) was the random forest. The intervention (random forest) performed better than the comparison (nearest neighbor) in terms of the accuracy: 62% vs. 49%. The top five SNPs for the prediction of asthma were rs7541950, rs7541956, rs7542025, rs7542028, and rs7542082. This study shows the usefulness of genetic artificial intelligence for the prediction of genetic predisposition to multifactorial diseases like asthma. However, few reviews are available on the recent progress of genetic artificial intelligence for the early diagnosis of GID. In this context, the purpose of this study is to conduct a systematic review for the recent progress of genetic artificial intelligence in GID.

## 2. Methods

### 2.1. Data and Search Terms

**Figure 2:**
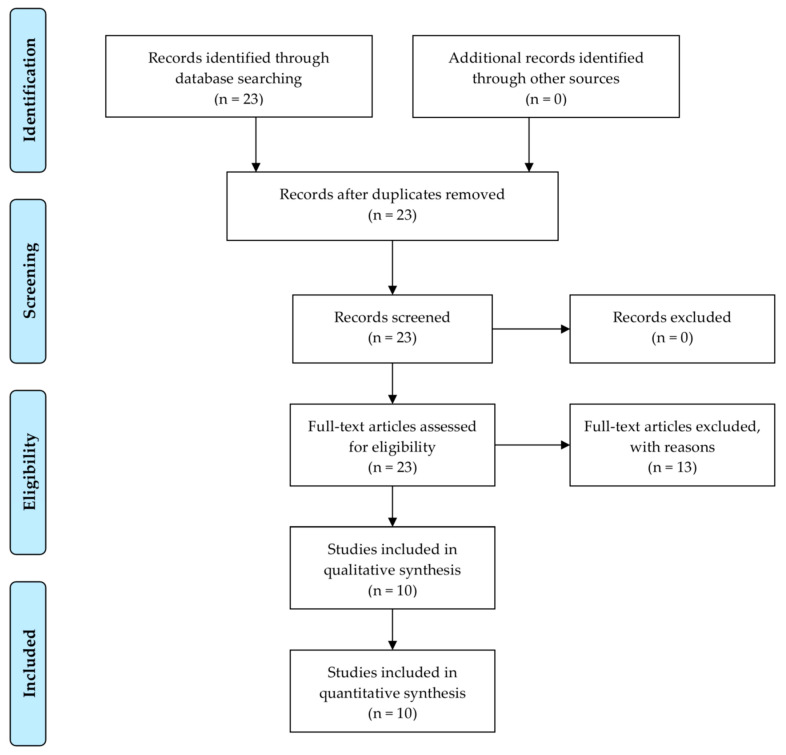
Flow diagram.

Figure 2 shows the flow diagram of this study. Data came from ten original studies from PubMed with the following search terms in titles or abstracts: (“snp*” or “single nucleotide polymorphism”) and (“gastro*” or “intestin*” or “diet*” or “digest*” or “stomach*”) and (“machine learning” or “neural network” or “random forest” or “deep learning” or “language model”). The search was conducted during 16 March 2025–15 April 2025.

### 2.2. Inclusion and Exclusion Criteria

The ten original studies were eligible according to the following criteria: participants with the dependent variable of GID or associated disease; interventions/comparisons of artificial intelligence; outcomes of accuracy, the area under the curve (AUC), and/or variable importance; a publication year of 2010 or later; and the publication language of English. Opinions and reviews were excluded.

### 2.3. Summary Measures

The following summary measures were adopted: (1) sample size (participants/cases), baseline vs. innovation artificial intelligence methods (comparisons vs. interventions), dependent variable (participants), task type; (2) baseline vs. innovation performance outcomes; (3) major demographic, health-related, and SNP predictors. Here, accuracy denotes the proportion of correct predictions over all observations. The area under the curve (AUC) represents the area under the plot of the true positive rate (sensitivity) against the false positive rate (1-specificity) at various threshold settings. The AUC is a major performance criterion in this study, given that it accommodates sensitivity and specificity.

## 3. Results

### 3.1. Summary

A summary of the review for the ten original studies [51,52,53,54,55,56,57,58,59,60] is presented in Table 2, Table 3 and Table 4. The “Study” column in the tables denotes the reference numbers of the ten original studies. The tables include (1) sample size (participants/cases), baseline vs. innovation artificial intelligence methods, dependent variable and task type (Table 2); (2) baseline vs. innovation performance outcomes (Table 3); (3) major demographic, health-related, and SNP predictors (Table 4). The performance outcomes reported were within 79–100 for accuracy (%) and 63–98 for the AUC (%). The random forest registered the best performance (AUC 98%) for the classifications of inflammatory bowel disease with 13 single nucleotide polymorphisms (SNPs). Likewise, the random forest delivered the best performance (R-square 99%) for the regression of the gut microbiome SNP saturation number. The following SNPs were discovered to be major predictors of GID or associated disease: rs2295778, rs13337626, rs2296188, rs2114039 (esophageal adenocarcinoma); rs28785174, rs60532570, rs13056955, rs7660164 (Crohn’s disease early intestinal resection); rs4945943 (Crohn’s disease); rs316115020, rs316420452 (calcium metabolism); rs738409_G, rs2642438_A, rs58542926_T, rs72613567_TA (steatotic liver disease); and rs148710154, rs75146099 (esophageal squamous cell carcinoma). The following demographic and health-related variables were found to be important predictors of GID or associated disease besides SNPs: age, body mass index, disease behavior, immune cell type, intestinal microbiome, MARCKS protein, smoking, and SNP density/number. No deep learning study was found even though deep learning was used as a search term together with machine learning. However, artificial intelligence is a data-driven approach, and more research is needed for more general conclusions.

### 3.2. Genetic Artificial Intelligence for Inflammatory Bowel Disease

This section describes three original studies of genetic artificial intelligence for inflammatory bowel disease [52,54,57]. A recent study used boosting as a predictive and explainable artificial intelligence technique for the classification of Crohn’s disease early intestinal dissection [52]. The data came from 463 participants enrolled in 15 general hospitals in Korea. The baseline approach (comparison) was baseline boosting (excluding SNPs) while the innovation approach (intervention) was genetic boosting (including SNPs). The intervention (genetic boosting) performed better than the comparison (baseline boosting) in terms of the AUC: 84% vs. 81%. Age and disease behavior were major features of genetic boosting together with four SNPs, i.e., rs28785174, rs60532570, rs13056955, and rs7660164. Another recent study adopted Linkage Disequilibrium (LD) as the predictive and explainable machine learning technique for the classification of Crohn’s disease [54]. The data source was 8421 cases enrolled in the European Genome-phenome Archive. The Least Absolute Shrinkage and Selection Operator (LASSO) served as the comparison whereas the LD served as the intervention. The intervention (LD) surpassed the comparison (LASSO) in terms of the AUC: 63% vs. 52%. One SNP (rs4945943) and MARCKS protein were important features of LD for the prediction of Crohn’s disease. Likewise, a recent study employed the random forest as a predictive and explainable machine learning technique for the classification of inflammatory bowel disease [57]. The data consisted of 757,042 cases enrolled in genome-wide association studies for European populations. The artificial neural network and the random forest were considered as the comparison and the intervention, respectively. The AUC of the intervention (random forest) was better than that of the comparison (artificial neural network), i.e., 98% vs. 91%. Thirteen SNPs (e.g., EXOC3: 6, SLC25A26: 1, YIF1B: 6) and intestinal microbiomes were significant features of the random forest for the prediction of inflammatory bowel disease.

### 3.3. Genetic Artificial Intelligence for Gastrointestinal Cancer

This section focuses on three original studies based on genetic artificial intelligence for gastrointestinal cancer [51,55,60]. A recent study used the random forest as a predictive and explainable artificial intelligence technique for the classification of esophageal adenocarcinoma [51]. The data came from 335 participants enrolled in a general hospital in the United States. The baseline approach (comparison) was the random forest including gastroesophageal reflux disease (RF-GERD) while the innovation approach (intervention) was the random forest including smoking (RF-Smoking). The intervention (RF-Smoking) went beyond the comparison (RF-GERD) in terms of the AUC, i.e., 80% vs. 70%. Besides smoking, nine SNPs were major predictors of RF-Smoking for esophageal adenocarcinoma: rs2295778, rs13337626, rs2296188, rs2114039, rs11941492, rs17708574, rs7324547, rs17619601, and rs17625898. Another recent study adopted the random forest as the predictive and explainable machine learning technique for the classification of colorectal cancer [55]. The data source was 26 cases enrolled in a general hospital in China. The random forest including microbiome (RF Microbiome Baseline) served as the comparison whereas the random forest including microbiome SNPs (RF Microbiome SNP) served as the intervention. The intervention (RF Microbiome SNP) surpassed the comparison (RF Microbiome Baseline) in terms of accuracy: 92% vs. 87%. Here, IB175794, BA459738, and EM8439 were found to be important intestinal microbiome SNPs. In a similar context, a recent study employed the random forest as the predictive and explainable machine learning technique for the classification of esophageal squamous cell carcinoma survival [60]. The data consisted of 439 participants enrolled in the Gene Expression Omnibus database. The random forest for 1-year survival and the random forest for 2-year survival were considered as the comparison and the intervention, respectively. The C-index of the intervention (random forest for 2-year survival) was beyond that of the comparison (random forest for 1-year survival), i.e., 80% vs. 65%. Two SNPs (rs148710154, rs75146099) and immune cell type were significant predictors of the random forest for esophageal squamous cell carcinoma survival.

### 3.4. Genetic Artificial Intelligence for Other Gastrointestinal Diseases

This section highlights three original studies based on genetic artificial intelligence for other gastrointestinal diseases [53,56,58]. A recent study used the random forest as the predictive and explainable artificial intelligence technique for the regression of gut microbiome SNP saturation number [53]. The data came from three participants enrolled in the European Nucleotide Archive. The baseline approach (comparison) was linear regression while the innovation approach (intervention) was the random forest. The intervention (random forest) was superior to the comparison (linear regression) in terms of the R-square: 99% vs. 80%. SNP density/number was a major variable of the random forest for the regression of gut microbiome SNP saturation number. Another recent study adopted the LASSO as the predictive and explainable machine learning technique for the classification of GI nematodes [56]. The data source was 1664 cases enrolled in Brazil. The artificial neural network served as the comparison whereas the LASSO served as the intervention. The intervention (LASSO) surpassed the comparison (artificial neural network) in terms of accuracy: 79% vs. 65%. A total of 41,676 SNPs were important variables of LASSO for the prediction of GI nematodes. In a similar vein, a recent study employed the random forest as the predictive and explainable machine learning technique for the classification of calcium metabolism [58]. The data consisted of 570 cases enrolled in China. The Wilcoxon test and random forest were considered as the comparison and the intervention, respectively. The accuracy of the intervention (random forest) was higher than that of the comparison (Wilcoxon’s test), i.e., 100% vs. 25%. Two SNPs (rs148710154, rs75146099) and intestinal microbiomes were significant variables of random forest for the prediction of calcium metabolism.

## 4. Discussion

### 4.1. Contributions of This Study

As addressed above, the application of predictive and explainable artificial intelligence to bioinformatics data such as SNP information is registering a rapid expansion in the diagnosis of various diseases [43,44,45,46,47,48,49,50]. However, little review has been conducted on the recent progress of genetic artificial intelligence for the early diagnosis of GID. In this vein, this study conducted a systematic review for the recent progress of genetic artificial intelligence in GID. One potential contribution of this review would be for pharmacology, which is the systematic study of drugs and their effects on living organisms. In other words, it focuses on the interactions between chemical and biological systems. It covers the chemical compositions, biological mechanisms, therapeutic uses, and potential toxicities of drugs. Here, SNPs can become drug targets or affect drug response for various diseases such as diabetes, obesity, and psychiatric disorders [61]. As addressed above, SNPs can serve as biomarkers, which distinguish those with disease from those without disease. Also, SNPs within transcription factor binding sites can influence the transcription rates of target genes thereby inducing a certain disease or its phenotypes [62]. This would be true for GID as well. SNPs can become drug targets or affect drug response based on GID proteomics and genomics.

### 4.2. Limitations of Existing Literature

Previous studies on the early diagnosis of GID based on explainable artificial intelligence had some limitations. Firstly, the four approaches of explainable artificial intelligence at this point—random forest impurity importance, random forest permutation importance, machine learning accuracy importance, and SHAP—can yield different results in certain circumstances. Random forest impurity importance can vary depending on how variables are categorized, while random forest permutation importance is relatively unaffected by this potential variation [21]. It should be noted, however, that random forest is unique in considering sequential information and this unique strength becomes more apparent with its impurity importance than with its permutation importance. In this vein, it is highly recommended to compare the four approaches of explainable artificial intelligence in a comprehensive manner. Secondly, it was beyond the scope of this review to consider other types of explainable artificial intelligence such as local interpretable model-agnostic explanations (LIME) [63]. Thirdly, the AUC of a study (0.63) [54] was below the optimal level for a diagnostic test.

### 4.3. Suggestions for Future Research

Some suggestions for this line of research are presented here. Firstly, uniting various modes of explainable artificial intelligence for various modes of GID data would give deeper clinical insights. For instance, one recent study [64] developed the random forest prediction system of oral cancer survival based on pathological (image), genetic, and clinical (numeric) data. The multi-modal random forest was far beyond other approaches in terms of model performance (c-index), i.e., 83% vs. multi-modal boosting (75%), multi-modal Cox (74%), clinical random forest (70%), genetic random forest (64%), and pathological random forest (64%). Here, it can be noted that clinical, genetic, and pathological predictors were equally important in unimodal models. In addition, protein binding was highly enriched based on gene enrichment analysis, whereas plasma membranes, secreted proteins, and extracellular regions were highly represented according to cellular component analysis. However, little literature is available, and more examination is needed regarding the combination of various modes of explainable artificial intelligence for various modes of GID data.

Secondly, more rigorous qualitative evaluation approaches are needed regarding systematic reviews of genetic artificial intelligence in GID. The Enhancing the Quality and Transparency of Health Research Network suggests the inclusion of a research question; eligibility and exclusion criteria; flow diagram; and experimental characteristics including sample size (participants), baseline vs. innovation methods (comparisons vs. interventions), dependent variable (participants), task type, baseline vs. innovation performance outcomes, and participant characteristics [65,66]. This study adopted the following suggestions: research question (Section 1); eligibility and exclusion criteria (Section 2); flow diagram (Figure 2); and experimental characteristics such as sample size, baseline vs. innovation artificial intelligence methods, dependent variable, task type (Table 2), baseline vs. innovation performance outcomes (Table 3), and major demographic, health-related, and SNP predictors (Table 4). But, more rigorous qualitative evaluation approaches can be introduced and this new guideline is likely to strengthen the validity of reviews for genetic artificial intelligence in GID.

Thirdly, little literature has been available and more investigation is needed on genetic artificial intelligence for reinforcement learning. Reinforcement learning is a division of artificial intelligence in which the environment gives a series of rewards, an agent responds with a series of actions to maximize the cumulative reward, and the environment makes a transition to the next period with given probabilities [67]. Reinforcement learning artificial intelligence begins like a human player with limited information in the limited periods available but eventually surpasses the best human player ever with the sheer power of big data (“temporal difference learning” in a professional language) [67]. Reinforcement learning has enjoyed great success in finance [68] and healthcare [69,70,71]. However, little research has been conducted and more examination is needed on explainable reinforcement learning. Reportedly, there have been a few studies in this direction and these studies have centered on simplified models with easy interpretation but insufficient performance and little consideration of the psychological and social factors behind optimization processes [72].

### 4.4. Conclusions

In summary, this study reviewed the recent advances in genetic artificial intelligence for the early diagnosis of GID. The performance results reported were within 79–100 for accuracy (%) and 63–98 for the AUC (%). Random forest presented the best performance (AUC 98%) for the classifications of inflammatory bowel disease with 13 single nucleotide polymorphisms (SNPs). Similarly, random forest showed the best performance (R-square 99%) for the regression of the gut microbiome SNP saturation number. The following SNPs were found to be major variables of GID or associated disease: rs2295778, rs13337626, rs2296188, rs2114039 (esophageal adenocarcinoma); rs28785174, rs60532570, rs13056955, rs7660164 (Crohn’s disease early intestinal resection); rs4945943 (Crohn’s disease); rs316115020, rs316420452 (calcium metabolism); rs738409_G, rs2642438_A, rs58542926_T, rs72613567_TA (steatotic liver disease); and rs148710154, rs75146099 (esophageal squamous cell carcinoma). The following demographic and health-related predictors were discovered to be important variables of GID or associated disease besides SNPs: age, body mass index, disease behavior, immune cell type, intestinal microbiome, MARCKS protein, smoking, and SNP density/number. In conclusion, genetic artificial intelligence is effective and non-invasive as a decision support system for GID.

## Figures and Tables

**Table 1:** RNA and DNA codons.

Amino Acid	RNA Codons	DNA Codons
Ala A	GCU, GCC, GCA, GCG	GCT, GCC, GCA, GCG
Arg R	CGU, CGC, CGA, CGG; AGA, AGG	CGT, CGC, CGA, CGG; AGA, AGG
Asn N	AAU, AAC	AAT, AAC
Asp D	GAU, GAC	GAT, GAC
Asn/Asp B	AAU, AAC; GAU, GAC	AAT, AAC; GAT, GAC
Cys C	UGU, UGC	TGT, TGC
Gln Q	CAA, CAG	CAA, CAG
Glu E	GAA, GAG	GAA, GAG
Gln/Glu Z	CAA, CAG; GAA, GAG	CAA, CAG; GAA, GAG
Gly G	GGU, GGC, GGA, GGG	GGT, GGC, GGA, GGG
His H	CAU, CAC	CAT, CAC
Ile I	AUU, AUC, AUA	ATT, ATC, ATA
Leu L	CUU, CUC, CUA, CUG; UUA, UUG	CTT, CTC, CTA, CTG; TTA, TTG
Lys K	AAA, AAG	AAA, AAG
Met M	AUG	ATG
Phe F	UUU, UUC	TTT, TTC
Pro P	CCU, CCC, CCA, CCG	CCT, CCC, CCA, CCG
Ser S	UCU, UCC, UCA, UCG; AGU, AGC	TCT, TCC, TCA, TCG; AGT, AGC
Thr T	ACU, ACC, ACA, ACG	ACT, ACC, ACA, ACG
Trp W	UGG	TGG
Tyr Y	UAU, UAC	TAT, TAC
Val V	GUU, GUC, GUA, GUG	GTT, GTC, GTA, GTG
Start	AUG, CUG, UUG	ATG, TTG, GTG, CTG
Stop	UAA, UGA, UAG	TAA, TGA, TAG

**Table 2:** Summary—sample size, method, and dependent variable.

Study	Sample Size	Method—Baseline	Method—Innovation	Dependent Variable	Type
[51]	335	RF GERD Included	RF Smoking Included	Esophageal Adenocarcinoma	Classification
[52]	463	Boosting SNP Excluded	Boosting SNP Included	Crohn’s Disease EIR	Classification
[53]	3	LR	RF	Gut Microbiome SNP SN	Regression
[54]	8421	LASSO	LD	Crohn’s Disease	Classification
[55]	26	RF Microbiome Baseline	RF Microbiome SNP	Colorectal Cancer	Classification
[56]	1664	ANN	LASSO	GI Nematode Resistance	Classification
[57]	757,042	ANN	RF	Inflammatory Bowel Disease	Classification
[58]	570	Wilcoxon Rank Sum	RF	Calcium Metabolism	Classification
[59]	199,732		Cox	Steatotic Liver Disease	Classification
[60]	439	RF 1-Year Survival	RF 2-Year Survival	ESCC	Classification

Note. Method: ANN—Artificial Neural Network; LASSO—Least Absolute Shrinkage and Selection Operator; LD—Linkage Disequilibrium; LR—Linear/Logistic Regression; RF—Random Forest; Dependent Variable: ESCC—Esophageal Squamous Cell Carcinoma; EIR—Early Intestinal Resection; GI—Gastrointestinal; SN—Saturation Number.

**Table 3:** Summary—model performance.

Study	Performance—Baseline		Performance—Comparison	
	Accuracy	Area Under the Curve	Accuracy	Area Under the Curve
[51]		70		80
[52]		81		84
[53]		80		99
[54]		52		63
[55]	87		92	
[56]	65		79	
[57]		91		98
[58]	25		100	
[59]				99
[60]		65		80
Min	25	52	79	63
Max	87	91	100	99
	R-Square			
	100 × (1 − *p* value)			

**Table 4:** Summary—major predictor.

Study	Predictor Demographic	Predictor Health	Predictor SNP			
[51]		GERD Smoking BMI	rs2295778	rs13337626	rs2296188	rs2114039
			rs11941492	rs17708574	rs7324547	rs17619601
			rs17625898			
[52]	Age	Disease Behavior	rs28785174	rs60532570	rs13056955	rs7660164
[53]		SNP Density/Number				
[54]		MARCKS Protein	rs4945943			
[55]		Microbiome Intestinal	IB175794	BA459738	EM8439	
[56]			SNPs 41676			
[57]		Microbiome Intestinal	SNPs 13	EXOC3: 6	SLC25A26: 1	YIF1B: 6
[58]		Microbiome Intestinal	rs316115020	rs316420452		
[59]			rs738409_G	rs2642438_A	rs58542926_T	rs72613567_TA
[60]		Immune Cell Types	rs148710154	rs75146099		

Note. Predictor Health: BMI—Body Mass Index; GERD—Gastroesophageal Reflux Disease; Predictor SNP (Single Nucleotide Polymorphism): BA—Blautia_A sp900066145; EM—Eubacterium_M sp; IB—Intestinibacter Bartlettii; SNP—Single Nucleotide Polymorphism.

## Data Availability

The original contributions presented in this study are included in the article. Further inquiries can be directed to the corresponding authors.

## References

[1] Cleveland Clinic Health: Gastrointestinal Diseases. https://my.clevelandclinic.org/health/articles/7040-gastrointestinal-diseases.

[2] Milivojevic V., Milosavljevic T. (2020). Burden of Gastroduodenal Diseases from the Global Perspective. Curr. Treat. Opt. Gastroenterol..

[3] Peery A.F., Crockett S.D., Murphy C.C., Jensen E.T., Kim H.P., Egberg M.D., Lund J.L., Moon A.M., Pate V., Barnes E.L. (2022). Burden and Cost of Gastrointestinal, Liver, and Pancreatic Diseases in the United States: Update 2021. Gastroenterology.

[4] Kim Y.-E., Park H., Jo M.-W., Oh I.-H., Go D.-S., Jung J., Yoon S.-J. (2019). Trends and Patterns of Burden of Disease and Injuries in Korea Using Disability-Adjusted Life Years. J. Korean Med. Sci..

[5] Jung H.-K., Jang B., Kim Y.H., Park J., Park S.Y., Nam M.-H., Choi M.-G. (2011). Health Care Costs of Digestive Diseases in Korea. Korean J. Gastroenterol..

[6] National Institute of Diabetes and Digestive and Kidney Diseases Digestive Diseases. https://www.niddk.nih.gov/health-information/digestive-diseases.

[7] Lee K.-S., Ahn K.H. (2020). Application of Artificial Intelligence in Early Diagnosis of Spontaneous Preterm Labor and Birth. Diagnostics.

[8] Ayana G., Park J., Jeong J.W., Choe S.W. (2022). A novel multistage transfer learning for ultrasound breast cancer image classification. Diagnostics.

[9] Zhang Y., Weng Y., Lund J. (2022). Applications of explainable artificial intelligence in diagnosis and surgery. Diagnostics.

[10] Heo J.S., Yang S.W., Lee S., Lee K.S., Ahn K.H. (2025). Sex-based differences in prenatal and perinatal predictors of autism spectrum disorder using machine learning with national health data. Autism Res..

[11] Lee S.J., Lee K.S., Park S.H., Lee S.W., Kim S.J. (2024). A machine learning-based decision support system for the prognostication of neurological outcomes in successfully resuscitated out-of-hospital cardiac arrest patients. J. Clin. Med..

[12] Saux P., Bauvin P., Raverdy V., Teigny J., Verkindt H., Soumphonphakdy T., Debert M., Jacobs A., Jacobs D., Monpellier V. (2023). Development and validation of an interpretable machine learning-based calculator for predicting 5-year weight trajectories after bariatric surgery: A multinational retrospective cohort SOPHIA study. Lancet Digit. Health.

[13] Kang K.W., Choi Y., Lim H.J., Kwak K., Choi Y.S., Park Y., Kim B.S., Lee K.S., Ahn K.H. (2025). Impact of platelet transfusion and bleeding risk stratification in patients with immune thrombocytopenia before procedures. Sci. Rep..

[14] Lee K.S., Ham B.J. (2024). Graph machine learning with systematic hyper-parameter selection on hidden networks and mental health conditions in the middle-aged and old. Psychiatry Investig..

[15] Lee S., Lee K.S. (2024). Predictive and explainable artificial intelligence for neuroimaging applications. Diagnostics.

[16] Park J.S., Lee K.S., Heo J.S., Ahn K.H. (2024). Clinical and dental predictors of preterm birth using machine learning methods: The MOHEPI study. Sci. Rep..

[17] Lee S., Lee E., Lee K.S., Pyun S.B. (2024). Explainable artificial intelligence on safe balance and its major determinants in stroke patients. Sci. Rep..

[18] Yang S.W., Lee K.S., Heo J.S., Choi E.S., Kim K., Lee S., Ahn K.H. (2024). Machine learning analysis with population data for prepregnancy and perinatal risk factors for the neurodevelopmental delay of offspring. Sci. Rep..

[19] Ma J., Xie Y. (2025). Machine learning techniques for independent gait recovery prediction in acute anterior circulation ischemic stroke. J. Neuroeng. Rehabil..

[20] R Package Randomforest. https://cran.r-project.org/web/packages/randomForest/randomForest.pdf.

[21] Python Package Sklearn. Ensemble. Random Forest Classifier. https://scikit-learn.org/stable/modules/generated/sklearn.ensemble.RandomForestClassifier.html.

[22] Lundberg S., Lee S.I. (2017). A unified approach to interpreting model predictions. arXiv.

[23] Python Package Shap. https://github.com/slundberg/shap.

[24] Mokhtari K.E., Higdon B.P., Basar A. Interpreting financial time series with SHAP values. Proceedings of the 29th Annual International Conference on Computer Science and Software Engineering.

[25] Mangalathu S., Hwang S.-H., Jeon J.-S. (2020). Failure mode and effects analysis of RC members based on machine-learning-based SHapley Additive exPlanations (SHAP) approach. Eng. Struct..

[26] Parsa A.B., Movahedi A., Taghipour H., Derrible S., Mohammadian A. (2020). (Kouros) toward safer highways, application of XGBoost and SHAP for real-time accident detection and feature analysis. Accid. Anal. Prev..

[27] Kha Q.-H., Le V.-H., Hung T.N.K., Le N.Q.K. (2021). Development and Validation of an Efficient MRI Radiomics Signature for Improving the Predictive Performance of 1p/19q Co-Deletion in Lower-Grade Gliomas. Cancers.

[28] Manikis G., Ioannidis G., Siakallis L., Nikiforaki K., Iv M., Vozlic D., Surlan-Popovic K., Wintermark M., Bisdas S., Marias K. (2021). Multicenter DSC–MRI-Based Radiomics Predict IDH Mutation in Gliomas. Cancers.

[29] Laios A., Kalampokis E., Johnson R., Munot S., Thangavelu A., Hutson R., Broadhead T., Theophilou G., Leach C., Nugent D. (2022). Factors predicting surgical effort using explainable artificial intelligence in advanced stage epithelial ovarian cancer. Cancers.

[30] Buergel T., Steinfeldt J., Ruyoga G., Pietzner M., Bizzarri D., Vojinovic D., zu Belzen J.U., Loock L., Kittner P., Christmann L. (2022). Metabolomic profiles predict individual multidisease outcomes. Nat. Med..

[31] Song S.I., Hong H.T., Lee C., Lee S.B. (2022). A machine learning approach for predicting suicidal ideation in post stroke patients. Sci. Rep..

[32] Kruk M., Goździejewska A.M., Artiemjew P. (2022). Predicting the effects of winter water warming in artificial lakes on zooplankton and its environment using combined machine learning models. Sci. Rep..

[33] Karim M.R., Islam T., Shajalal M., Beyan O., Lange C., Cochez M., Rebholz-Schuhmann D., Decker S. (2023). Explainable AI for bioinformatics: Methods, tools and applications. Brief Bioinform..

[34] Vilhekar R.S., Rawekar A. (2024). Artificial Intelligence in Genetics. Cureus.

[35] Claverie J.M., Cedric Notredame C. (2007). Bioinformatics for Dummies.

[36] Pevsner J. (2015). Bioinformatics and Functional Genomics.

[37] National Center for Biotechnology Information GenBank. https://www.ncbi.nlm.nih.gov/genbank/about/.

[38] Weizmann Institute of Science GeneCards. https://www.genecards.org/.

[39] European Molecular Biology Laboratory-European Bioinformatics Institute UniProt. https://www.uniprot.org/.

[40] Genetic Education Inc. What Is Single Nucleotide Polymorphism (SNP)?. https://geneticeducation.co.in/what-is-single-nucleotide-polymorphism-snp/.

[41] National Human Genome Research Institute Single Nucleotide Polymorphisms (SNPs). https://www.genome.gov/genetics-glossary/Single-Nucleotide-Polymorphisms-SNPs.

[42] Media Wiki and National Center for Biotechnology Information. https://www.snpedia.com/index.php/SNPedia.

[43] Gaudillo J., Rodriguez J.J.R., Nazareno A., Baltazar L.R., Vilela J., Bulalacao R., Domingo M., Albia J. (2019). Machine learning approach to single nucleotide polymorphism-based asthma prediction. PLoS ONE.

[44] López B., Torrent-Fontbona F., Viñas R., Fernández-Real J.M. (2018). Single Nucleotide Polymorphism relevance learning with Random Forests for Type 2 diabetes risk prediction. Artif. Intell. Med..

[45] Muflikhah L., Fatyanosa T.N., Widodo N., Perdana R.S., Solimun, Ratnawati H. (2025). Feature selection for hypertension risk prediction using XGBoost on single nucleotide polymorphism data. Healthc. Inform. Res..

[46] Tai K.Y., Dhaliwal J., Wong K. (2022). Risk score prediction model based on single nucleotide polymorphism for predicting malaria: A machine learning approach. BMC Bioinform..

[47] Ameli A., Peña-Castillo L., Usefi H. (2024). Assessing the reproducibility of machine-learning-based biomarker discovery in Parkinson’s disease. Comput. Biol. Med..

[48] Aguiar-Pulido V., Seoane J.A., Rabuñal J.R., Dorado J., Pazos A., Munteanu C.R. (2010). Machine learning techniques for single nucleotide polymorphism-disease classification models in schizophrenia. Molecules.

[49] Silva P.P., Gaudillo J.D., Vilela J.A., Roxas-Villanueva R.M.L., Tiangco B.J., Domingo M.R., Albia J.R. (2022). A machine learning-based SNP-set analysis approach for identifying disease-associated susceptibility loci. Sci. Rep..

[50] Ghorbani A., Rostami M., Guzzi P.H. (2024). AI-enabled pipeline for virus detection, validation, and SNP discovery from next-generation sequencing data. Front. Genet..

[51] Zhai R., Zhao Y., Liu G., Ter-Minassian M., Wu I.C., Wang Z., Su L., Asomaning K., Chen F., Kulke M.H. (2012). Interactions between environmental factors and polymorphisms in angiogenesis pathway genes in esophageal adenocarcinoma risk: A case-only study. Cancer.

[52] Kang E.A., Jang J., Choi C.H., Kang S.B., Bang K.B., Kim T.O., Seo G.S., Cha J.M., Chun J., Jung Y. (2021). Development of a Clinical and Genetic Prediction Model for Early Intestinal Resection in Patients with Crohn’s Disease: Results from the IMPACT Study. J. Clin. Med..

[53] Liu P., Hu S., He Z., Feng C., Dong G., An S., Liu R., Xu F., Chen Y., Ying X. (2022). Towards Strain-Level Complexity: Sequencing Depth Required for Comprehensive Single-Nucleotide Polymorphism Analysis of the Human Gut Microbiome. Front. Microbiol..

[54] Garza-Hernandez D., Estrada K., Trevino V. (2022). Multivariate genome-wide association study models to improve prediction of Crohn’s disease risk and identification of potential novel variants. Comput. Biol. Med..

[55] Han S., Zhuang J., Pan Y., Wu W., Ding K. (2022). Different Characteristics in Gut Microbiome between Advanced Adenoma Patients and Colorectal Cancer Patients by Metagenomic Analysis. Microbiol. Spectr..

[56] Freitas L.A., Savegnago R.P., Alves A.A.C., Stafuzza N.B., Pedrosa V.B., Rocha R.A., Rosa G.J.M., Paz C.C.P. (2024). Genome-enabled prediction of indicator traits of resistance to gastrointestinal nematodes in sheep using parametric models and artificial neural networks. Res. Vet. Sci..

[57] Bi G.W., Wu Z.G., Li Y., Wang J.B., Yao Z.W., Yang X.Y., Yu Y.B. (2024). Intestinal flora and inflammatory bowel disease: Causal relationships and predictive models. Heliyon.

[58] Jin J., Li Q., Zhou Q., Li X., Lan F., Wen C., Wu G., Li G., Yan Y., Yang N. (2024). Calcium deposition in chicken eggshells: Role of host genetics and gut microbiota. Poult. Sci..

[59] Schophaus S., Creasy K.T., Koop P., Clusmann J., Jaeger J., Punnuru V., Koch A., Trautwein C., Loomba R., Luedde T. (2024). Machine learning uncovers manganese as a key nutrient associated with reduced risk of steatotic liver disease. Liver Int..

[60] Wang H., Ma S., Yang Z., Niu R., Zhu H., Li S., Gao S., Li Z., Tian Y. (2025). Revolutionizing ESCC prognosis: The efficiency of tumor-infiltrating immune cells (TIIC) signature score. Discov. Oncol..

[61] Ahmed I., Siddiqui H.I., Qureshi G.S., Bernhardt G.V. (2022). A Review of Literature on the Pharmacogenomics of Single-Nucleotide Polymorphisms. Biomed. Biotechnol. Res. J..

[62] Ramírez A.V.R., Farías A.F.-S., Álvarez R.d.C.C., de Oca E.P.M. (2021). Predicted regulatory SNPs reveal potential drug targets and novel companion diagnostics in psoriasis. J. Transl. Autoimmun..

[63] Ribeiro M.T., Singh S., Guestrin C. (2016). Why should I trust you? Explaining the predictions of any classifier. arXiv.

[64] Vollmer A., Hartmann S., Vollmer M., Shavlokhova V., Brands R.C., Kübler A., Wollborn J., Hassel F., Couillard-Despres S., Lang G. (2024). Multimodal artificial intelligence-based pathogenomics improves survival prediction in oral squamous cell carcinoma. Sci. Rep..

[65] Enhancing the Quality and Transparency of Health Research Network (2024). Reporting Guidelines. https://www.equator-network.org/reporting-guidelines/ten-simple-rules-for-neuroimaging-meta-analysis/.

[66] Müller V.I., Cieslik E.C., Laird A.R., Fox P.T., Radua J., Mataix-Cols D., Tench C.R., Yarkoni T., Nichols T.E., Turkeltaub P.E. (2018). Ten simple rules for neuroimaging meta-analysis. Neurosci. Biobehav. Rev..

[67] Majid Ghasemi M., Dariush Ebrahimi D. (2024). Introduction to reinforcement learning. arXiv.

[68] Hambly B., Xu R., Yang H. (2022). Recent advances in reinforcement learning in finance. arXiv.

[69] Yu C., Liu J., Nemati S. (2020). Reinforcement learning in healthcare: A survey. arXiv.

[70] Coronato A., Naeem M., De Pietro G., Paragliola G. (2020). Reinforcement learning for intelligent healthcare applications: A survey. Artif. Intell. Med..

[71] Liu S., See K.C., Ngiam K.Y., Celi L.A., Sun X., Feng M. (2020). Reinforcement learning for clinical decision support in critical care: Comprehensive review. J. Med. Internet Res..

[72] Puiutta E. (2020). Veith EMSP. Explainable reinforcement learning: A survey. arXiv.

